# Assessment of primary health care received by the elderly and health related quality of life: a cross-sectional study

**DOI:** 10.1186/1471-2458-13-605

**Published:** 2013-06-24

**Authors:** Vivian C Honorato dos Santos de Carvalho, Sinara L Rossato, Flávio D Fuchs, Erno Harzheim, Sandra C Fuchs

**Affiliations:** 1 Postgraduate Studies Program in Epidemiology, School of Medicine, Universidade Federal do Rio Grande do Sul, Ramiro Barcelos 2600, Porto CEP, 90035-003, Alegre, RS, Brazil; 2 National Institute for Science and Technology for Health Technology Assessment, (IATS), Hospital de Clinicas de Porto Alegre, Ramiro Barcelos 2350, Clinical Research Center, CEP, 90035–003, Porto Alegre, RS, Brazil

**Keywords:** Primary health care, Elderly, Quality of life, Family health, Family health strategy, Hypertension, Family medicine

## Abstract

**Background:**

Population aging leads to increased burden of chronic diseases and demand in public health. This study aimed to assess whether the score of Primary Health Care (PHC) is associated with a) the model of care - Family Health Strategy (FHS) *vs*. traditional care model (the Basic Health Units; BHU); b) morbid conditions such as - hypertension, diabetes mellitus, mental disorders, chronic pain, obesity and central obesity; c) quality of life in elderly individuals who received care in those units.

**Methods:**

A survey was conducted among the elderly between August 2010 and August 2011, in Ilheus, Bahia. We interviewed elderly patients - 60 years or older - who consulted at BHU or FHS units in that day or participated in a group activity, and those who were visited at home by the staff of PHC, selected through a random sample. Demographic and socioeconomic characteristics, services’ attainment of primary care attributes, health problems and quality of life were investigated. The Short Form Health Survey (SF-12) was used to assess quality of life and PCATool to generate PHC scores. In addition, weight, height and waist circumference were measured. Trained research assistants, under supervision performed the data collection.

**Results:**

A total of 511 elderly individuals were identified, two declined to participate, resulting in 509 individuals interviewed. The health care provided by the FHS has higher attainment of PHC attributes, in comparison to the BHU, resulting in lower prevalence of score below six. Except for hypertension and cardiovascular disease, other chronic problems were not independently associated with low scores in PHC. It was observed an independent and positive association between PHC score and the mental component of quality of life and an inverse association with the physical component.

**Conclusions:**

This study showed higher PHC attributes attainment in units with FHS, regardless of the health problem. The degree of orientation to PHC increased the mental component score of quality of life.

## Background

The trends toward reduce fertility rates and increase life expectancy at birth doubled the elderly population of Brazil from 1900 to 1989, and it is estimated that by 2050 the population aged 60 years or more will reach 22% [1]. The longevity of the elderly population has raised the demand for health care services [2], particularly those provided in Primary Health Care (PHC) [3].

In Brazil, the National Health System (SUS, Unified Health System) is based on universal free access to healthcare to the whole population, with decentralization provided at all levels – from prevention to high complexity level, shared by government, federal, state and municipal. The SUS includes the model of foresight system, which provides care in basic health units (BHUs) for spontaneous demand or patients referred by other services. The organization and provision of services are based on risk groups or specific conditions. In these units, the health care team usually includes internal medicine physicians, pediatricians and gynecologists, nurses and other health professionals. The target is not focused on the integration of health care [4], but in raising the level of health through reduction and control of the burden of disease in the population. These actions are developed in programs for women, children, adults and elderly, or focused on specific conditions.

The second model of PHC, based on Family Health Strategy (FHS), offers participative and comprehensive care, and a system of centralized coordination [4]. The FHS began in the poorest areas without coverage of PHC and since then has been implemented gradually replacing the traditional model of care. This model aims to insert the individual in the system, providing a primary person-centered care, with priority given to prevention and health promotion, and secondly for curative medicine. Coverage is universal, geographically circumscribed and referral to other levels of care are allowed when necessary [5]. The FHS involves health care implemented by nuclear teams, including a family doctor, a nurse, four community health workers, and other assistants. Each team is responsible for 1,000 families, up to 4500 individuals, [6] and family doctors should live in the communities where they work, [7] but this does not happen in Brazil.

The management of chronic diseases in Brazil [8] takes into account not only the prevalence, but also the population aging and the burden of disease [3]. While caring for the elderly can be done in any model [9], FHS or BHU, the guideline recommends easy access to SUS [10], with priority attention and frequency of assistance according to the presence of risk factors and health status throughout life, tracking changes and stages of aging, integrating promotion, prevention, treatment and rehabilitation at all levels of complexity of the healthcare system [4]. Health promotion for the elderly, in PHC is still incipient in Brazil and has scarce evaluation of effectiveness [11,12]. Among the assessment tools available, there is the Primary Care Assessment Tool (PCATool), validated in Brazil [13,14], that verifies the presence and extent of the essential and derivatives attributes of PHC [15-17], which allow analysis of structure and process [13]. The scores quantify the performance of PHC, taking into account the essential attributes and derivatives: the first contact for access, longitudinally, coordination, comprehensiveness, family-centered care and community-oriented [15].

Evaluations of the effectiveness of PHC model [17,18] have been performed in adults [19] and children [20,21], or from the perspective of the professionals [9,10], showing that FHS was superior to other options to achieve the goals set for the PHC. However, no study in the elderly has described the attributes of the PHC with FHS versus the traditional model of BHU.

PHC should be prepared to deal with highly prevalent morbid conditions. However, health centers located in deprived areas had low performance in terms of quality of care [22] and units with FHS are concentrated in poor areas. Moreover, the management of chronic diseases such as hypertension and diabetes mellitus, could be affected by the model of care. [23-25] Thus, quantifying the scores of PHC can not only check whether the objectives are being met, as they provide information to develop and implement alternatives to existing policies, ensuring better health and quality of life. This study aimed to compare the degree of person centered care (PHC score) in two different ways of providing primary care in Brazil (Family Health Strategy *vs*. Basic Health Units); to assess the association of hypertension, diabetes mellitus, mental disorders, chronic pain, obesity, and central obesity with PHC score; and to evaluate the PHC score with quality of life in elderly individuals who received care in those units.

## Methods

### Study design and participants

This cross-sectional study enrolled patients 60 years or older from units of Primary Health Care in Ilheus, Bahia, in northeastern Brazil, between August 2010 and August 2011. The city has 33 health units, 23 with FHS and UBS 10. Among those, 13 and 8 units, respectively, were selected by stratified random sampling, proportional to the number of units of each model of care. The units were visited by the supervisor in order to obtain consent to perform data collection. Research assistants assessed the eligibility of patients - 60 years or older - who were at the unit in that day, to consult or participate in a group activity, and for those who were visited at home by the staff of PHC.

The Ethical Committee of the Hospital Nossa Senhora da Conceição, from Porto Alegre, accredited by the Office of Human Research Protections the Institutional Review Board, approved the project (registry: GHC 090 090/09) and all participants signed a consent form.

### Studied variables

The main exposures were the model of care: UBS and FHS, followed by chronic conditions reported among major health problems: hypertension, diabetes mellitus, mental illness or chronic pain. Patient could report up to three diseases or symptoms in addition to abdominal obesity and body mass index. The clinical outcomes include attributes evaluated by PCATool answered by the patient. PCATool was used to detect the extent of four essential attributes: access to first contact, longitudinally, completeness and coordination, and two derived attributes: family and community orientation. A PCATool with cut-off of 6.6 or greater was considered high orientation. The second outcome was quality of life as assessed by the Short Form Health Survey, which includes 12 items, generating scores summarized by two components: physical (PCS, Physical Component Summary) and mental (MCS Mental Component Summary) [26]. Demographic variables (gender and age) and socioeconomic status (education, measured by years of education, work status and family life at home) were considered as potential confounders. The SF-12, version 2 [27], and PCATool [13,14], were both validated for Portuguese.

### Data collection

Trained research assistants conducted interviews at the unit or the elderly home using standardized questionnaires including demographic variables (age and sex), socioeconomic (education, in years, work status, marital status, family members living in the household), reported major health problems (hypertension, diabetes mellitus, mental disorders, chronic pain), and measured morbidity (general obesity and abdominal obesity). Anthropometry was performed in duplicate and the average was used for analysis. Waist circumference (cm) was measured at the midpoint between the lower rib margin and the superior iliac crest, with inelastic tape. Abdominal obesity was determined by waist circumference greater than 88 cm, for women, and 102 cm, for men. Weight (kg) was measured while the patient was balanced on both feet, with arms hanging freely, wearing light clothing and no shoes, with Techline scale (model BAL-180-CI, with 100 g precision). Height (m) was measured with Sanny portable stadiometer; weight and height were used to calculate body mass index (weight, in kilograms, divided by the square of height, in meters), categorized below 25.0 (reference category), overweight for 25.0 to 29.9, and obesity for 30.0 kg/m^2^ or higher. The research team underwent training, and the project implementation was tested in a pilot study with 30 individuals. Approximately 46% of interviews were conducted under supervision.

### Sample size calculation and statistical analysis

Considering the lack of data for elderly, the calculation was based on an estimate that 90% of BHUs would present low PHC score versus 75% of units with FHS, resulting in a sample of 352 participants to detect a prevalence ratio of at least 1.2, with P value of 0.05 (two tailed), and power of 90%. The sample size calculation was increased by 25% to maintain statistical power due to losses. Data were entered into the database, created in the Epinfo software, version 3.5.3 (CDC, Atlanta, GA, USA) and the analyzes were performed using the Statistical Package for Social Sciences, version 17.0 (SPSS Inc., Chicago, IL, USA). The chi-square test was used to compare proportions and analysis of variance (ANOVA) to test differences between means. Poisson regression - an alternative to analysis of binary outcomes in cross-sectional studies - assigning a constant at risk time for all participants provides risk ratios equivalent to prevalence ratios. Since the variance of the coefficients tends to be overestimated, which result in higher confidence intervals, robust variance estimators was used [28]. Modified Poisson regression was performed to assess independent associations and to calculate adjusted prevalence ratios with 95%CI for reported major health problems, type of PHC model, and low score of PHC. Multiple linear regression was used to assess the association between PHC score and quality of life domains adjusting for different covariate according to different regression models.

## Results

A total of 511 elderly individuals were identified, two declined to participate, resulting in 509 individuals enrolled. Table 1 presents the characteristics of participants according to the model of care, highlighting the overall predominance of women, aged 72.8 ± 8.2 years, with 13% living alone or only with a spouse and 3.5% were retired, but still working. More patients (19%) with higher education consulted at BHUs than in FHS (8.8%), while those who lived alone or only with spouse more often were patients of units with FHS (16 *vs*. 11%). The frequency of major health problems was similar in both models of care, but abdominal obesity and chronic pain were more frequent among patients who consulted in units with FHS. The score for quality of life for the physical component summary was higher in the BHU while the mental component summary was higher in the FHS.

**Table 1:** **Characteristics of participants evaluated in Primary Health Care units according to model of care**, **Ilheus**, **Bahia**, **Brazil** [**N** (%) **or mean** ±**SD**]

	**Total**	**Basic health unit**	**Family health strategy**	**P value**
	**n=509**	**n=316**	**n=193**	
Female sex	327 (64.2)	212 (67.1)	115 (59.6)	0.09
Age (years)	72.8 ± 8.2	72.7 ± 8.1	72.9 ± 8.4	0.8
Years at school				0.001
0	241 (47.3)	134 (42.4)	107 (55.4)	
1-4	190 (37.3)	121 (38.3)	64 (35.8)	
≥5	78 (15.3)	61 (19.3)	17 (8.8)	
Work status				0.8
Never worked	70 (13.8)	44 (13.9)	26 (13.5)	
Retaired	407 (80.0)	254 (80.4)	153 (79.3)	
Working	14 (2.8)	9 (2.8)	5 (2.6)	
Retaired, but working	18 (3.5)	9 (2.8)	9 (4.7)	
Family living in the household				0.002
Alone	67 (13.2)	36 (11.4)	31 (16.1)	
Spouse	118 (23.2)	62 (19.6)	56 (29.0)	
Spouse and family	110 (21.6)	83 (26.3)	27 (14.0)	
Extensive family or others	214 (42.0)	135 (42.7)	79 (40.9)	
Body mass index (kg/m^2^)*				0.8
<25.0	207 (40.7)	129 (41.0)	78 (40.4)	
25.0-29.9	179 (35.2)	108 (34.3)	71 (36.8)	
≥ 30.0	122 (24.0)	78 (24.8)	44 (22.8)	
Abdominal obesity				0.05
No	289 (56.8)	190 (60.1)	99 (51.3)	
Yes	220 (43.2)	126 (39.9)	94 (48.7)	
Reported major health problems				
Hypertension	144 (28.3)	93 (29.4)	51 (26.4)	0.5
Diabetes mellitus	67 (13.2)	45 (14.2)	22 (11.4)	0.4
Cardiovascular disease	27 (5.3)	16 (5.1)	11 (5.7)	0.8
Mental disorder	9 (1.8)	7 (2.2)	2 (1.0)	0.3
Chronic pain	127 (25.0)	65 (20.6)	62 (32.1)	0.003
Number of chronic conditions				0.13
0	59 (11.6)	43 (13.6)	16 (8.3)	
1	382 (75)	235 (74.4)	147 (76.2)	
≥2	68 (13.4)	38 (12.0)	30 (15.5)	
SF-12				
Physical component				
Summary	38.1 ± 11.6	39.0 ± 11.8	36.5 ± 11.2	0.02
Mental component				
Summary	48.7 ± 10.4	48.0 ± 9.5	49.9 ± 11.7	0.05

* One elderly was not able to stand up for measurement of height.

Table 2 shows that the scores of PHC for attributes such as affiliation, essential and overall were higher in the FHS, compared to BHU. The prevalence of higher scores was higher in the FHS model than in units with BHU.

**Table 2:** **Overall score**, **essential**, **and attributes to Primary Health Care** (**PHC**) **and score of PHC according to the model of care**, **Ilheus**, **Bahia**, **Brazil** [**mean** ±**SD**]

	**Basic health unit**^**1 **^**n=316**	**Family health strategy**^**2 **^**n=193**	**P value**
Access - utilization	5.2 ± 4.7	6.9 ± 4.2	<0.001
Access - accessibility	2.3 ± 2.6	3.7 ± 2.7	<0.001
Longitudinally	4.0 ± 4.2	6.5 ± 4.0	<0.001
Integrality	2.9 ± 1.7	3.5 ± 2.6	0.003
Coordination	2.8 ± 4.3	4.8 ± 4.9	0.001
Family orientation	1.5 ± 3.1	3.0 ± 4.3	<0.001
Community orientation	3.6 ± 3.5	4.3 ± 3.3	0.02
Essential	4.1 ± 2.2	5.5 ± 2.3	<0.001
Overall	3.6 ± 1.9	5.0 ± 2.1	<0.001

Table 3 presents the prevalence and characteristics associated with low score of PHC among the elderly. Low PHC score was more prevalent among elderly patients who were not working, had consulted at the UBS, with no hypertension, and had cardiovascular disease, regardless of demographic, socioeconomic characteristics, and health problems. Elderly patients who reported hypertension were less likely to have low PHC scores, even after controlling for education, work status and other confounding factors.

**Table 3:** **Prevalence and characteristics associated to low Primary Health Care score among elderly**, **Ilheus**, **Bahia**, **Brazil**

	**Low primary health care score**		
	**Prevalence**^**‡ **^**(%)**	**Prevalence ratio**^ƒ^* (95%CI)	**Prevalence ratio**^ƒ^** (95%CI)
Age (years)			
60-69	84 (84.8)	1.00	1.00
70-79	177 (86.3)	0.97 (0.91-1.05)	0.97 (0.91-1.04)
80-103	180 (87.8)	0.96 (0.87-1.05)	0.96 (0.87-1.05)
P value	0.8	0.6	0.6
Sex			
Male	157 (86.3)	1.00	1.00
Female	284 (86.9)	0.98 (0.91-1.05)	0.98 (0.91-1.05)
P value	0.9	0.5	0.5
Years at school			
0	203 (84.2)	0.98 (0.89-1.07)	0.97 (0.89-1.06)
1-4	168 (88.4)	1.02 (0.93-1.11)	1.01 (0.92-1.10)
≥5	70 (89.7)	1.00	1.00
P value	0.3	0.6	0.6
Work status			
Yes	22 (68.8)	1.00	1.00
No	419 (87.8)	1.29 (1.02-1.63)	1.28 (1.02-1.62)
P value	0.002	0.03	0.04
Family living in the household			
Alone	56 (83.6)	0.98 (0.87-1.10)	0.98 (0.87-1.09)
Spouse	103 (87.3)	1.02 (0.93-1.11)	1.01 (0.93-1.10)
Spouse and family	97 (88.2)	0.99 (0.90-1.07)	0.97 (0.89-1.06)
Extensive family or others	185 (86.4)	1.00	1.00
P value	0.8	0.9	0.8
Model of care			
Family Health Strategy	148 (76.7)	1.00	1.00
Basic Health Unit	293 (92.7)	1.20 (1.11-1.31)	1.21 (1.11-1.31)
P value	<0.001	<0.001	<0.001
Body mass index (kg/m^2^)			
<25.0	178 (86.0)	1.00	1.00
25.0-29*.*9	153 (85.5)	1.00 (0.92-1.08)	1.00 (0.93-1.08)
≥30.0	109 (89.3)	1.03 (0.95-1.11)	1.03 (0.95-1.12)
P value	0.6	0.7	0.8
Abdominal obesity			
No	255 (88.2)	1.00	1.00
Yes	186 (84.5)	0.97 (0.90-1.04)	0.96 (0.90-1.04)
P value	0.2	0.3	0.3
Hypertension			
No	324 (88.8)	1.00	1.00
Yes	117 (81.3)	0.91 (0.84-0.99)	0.92 (0.84-1.00)
P value	0.03	0.03	0.049
Diabetes mellitus			
No	381 (86.2)	1.00	1.00
Yes	60 (89.6)	1.02 (0.94-1.11)	1.03 (0.94-1.12)
P value	0.5	0.6	0.5
Cardiovascular disease			
No	414 (85.9)	1.00	1.00
Yes	27 (100.0)	1.17 (1.11-1.23)	1.15 (1.09-1.21)
P value	0.04	<0.001	<0.001
Mental disorder			
No	433 (86.6)	1.00	1.00
Yes	8 (88.9)	0.99 (0.81-1.22)	0.98 (0.79-1.20)
P value	0.8	0.9	0.8
Chronic pain			
No	332 (86.9)	1.00	1.00
Yes	109 (85.8)	1.03 (0.95-1.11)	1.01 (0.94-1.10)
P value	0.8	0.5	0.7
Number of chronic conditions			
0	92 (90.2)	1.00	1.00
1	203 (86.0)	0.98 (0.91-1.06)	1.00 (0.92-1.09)
≥2	146 (85.4)	0.97 (0.89-1.06)	1.00 (0.90-1.10)
P value	0.5	0.8	1.0

^‡^ Non-adjusted prevalence rate.

^ƒ^ Modified Poisson regression.

* Prevalence ratio adjusted for age, sex, years at school, working status, model of care

** Prevalence ratio adjusted for age, sex, years at school, working status, model of care, hypertension, cardiovascular disease.

Table 4 shows the independent association between the PHC scores and quality of life among elderly. There was a positive association between the score of the mental component of quality of life, while for the physical component, there was a negative association. The estimates did not change substantially after the control for confounding.

**Table 4:** **Association of Primary Health Care score with Physical and Mental Component Summary of quality of life**^ƒ^**among elderly**, **Ilheus**, **Bahia**

**Primary health care score**	**Mental component summary**		**Physical component summary**	
	**β coefficient (95% CI)**	**P value**	**β coefficient (95% CI)**	**P value**
No adjustment	0.085 (-0.008 to 0.876)	0.05	-0.123 (-1.191 to -0.209)	0.005
Model 1*	0.090 (0.015 to 0.895)	0.04	-0.124 (-1.189 to -0.222)	0.004
Model 2**	0.093 (0.032 to 0.914)	0.04	-0.115 (-1.133 to -0.170)	0.008
Model 3***	0.089 (0.008 to 0.893)	0.046	-0.129 (-1.216 to -0.251)	0.003

^ƒ^ Multiple linear regression.

* Adjusted for age and sex.

** Additionally adjusted for years at school.

*** Additionally adjusted for hypertension, and cardiovascular disease.

## Discussion

The assessment of PHC attributes attainment using PCATool among elderly individuals identified that those who consulted in units with FHS had higher PHC scores than those attending to BHU. Such association between model of care and PHC score was independent of socioeconomic status or major health problems. Work status, a surrogated marker of income, played a role in the adherence to PHC orientation and those elderly patients not working were more likely to have low score. Low PHC score was associated with two morbid conditions - hypertension (inversely) and cardiovascular disease (positively). Regardless of the health problem, the PHC scores were directly associated with the mental component of quality of life and negatively with the physical component. These results suggest that the model of care in PHC was associated with quality of life.

Evaluation of the PHC attributes attainment using PCATool [17,18,21] had already been done, but not for elderly patients. The higher PHC scores observed among elderly who consulted at FHS could be anticipated, since this model of care was planned to fulfill these attributes while BHUs aimed to a different target. Even so, is reassuring to detect that the FHS reached its purpose in elderly from Ilheus, Bahia, in Northeast Brazil. However, there are other dimensions to be assessed in the PHC. For instance, patient-physician relationship and teaching activities were positively associated with quality of care in a study conducted in the Catalan Primary Health Care - with universal coverage used by 75% of the population, which is quite similar to the Brazilian coverage by the national health system. In the Spanish study items such as accessibility and doctor-patient relationship were higher in rural areas, less privileged populations, and among teams involved in the care of elderly [29]. Although PCATooL and other instruments used to evaluate the attributes of PHC do not incorporate the doctor-patient relationship and adoption of evidence-based practices, they included the core attributes [30].

FHS units are located in more deprived areas than BHU units. This condition did not account for overall differences between elderly patients who consulted in both models of care. While mean age, work status, and morbid conditions - hypertension, diabetes mellitus, obesity, cardiovascular disease, and mental disorder – were similar among patients from both models of care, those who consulted in the FHS units were more likely to live alone or with a spouse, had low education level, higher prevalence of central obesity and chronic pain. These conditions were directly associated to the use of health care facilities [31,32]. In a multivariate analysis, the low PHC score was also associated with work status.

The report of morbid conditions as major health problems is not equivalent to the prevalence of these conditions. Some chronic conditions such as hypertension [33] and chronic pain [34], were underestimated. For instance, hypertension prevalence affected 68.9% (95% CI 64.1%–73.3%) of the Brazilian elderly population, according to the JOINT definition and using blood pressure measurement, 49.0% (95% CI 46.8%–51.2%) by self-reported in household surveys, or 53.8% (95% CI 44.8%–62.6%) by telephone surveys [35]. These numbers are far higher than the 30% of elderly reporting hypertension as a major health problem. Besides the label of being a hypertensive patient and taking lowering blood pressure medicine daily, hypertension does not cause symptoms and is easily underestimated as a burden of disease. Elderly with hypertension seems to benefit of home visiting and reinforcement to take blood pressure-lowering medication [23], resources offered in units with FHS. Its inverse association with low PHC score, independently of other confounding factors, might be attributed to characteristics of hypertension that demands frequent appointments for dispensing medicine and checking hypertension control.

Quality of life for the elderly population was shown to be associated with the PHC score, independent of confounding factors and even of hypertension, which is one of the determinants of reduced quality of life [33]. Morbid conditions can reduce health and, in turn, the physical component of quality of life [36,37]. Studies conducted in China [31] and Germany [32] have shown that low quality of life markedly increased the use of health services, but this relationship was characterized for PHC without comparison with traditional Chinese medicine or other type of healthcare. In the elderly, the deterioration of quality of life mainly due to the physical functioning rather than mental [38] was shown through the opposite association with PHC score. The inverse association between PHC score and physical component of quality of life in the elderly suggests the difficulty to benefit from health care system due to loss of physical functioning. Findings in elderly living in Australia showed a negative association of PCS-12 with characteristics of the practice, which highlighted the role of chronic diseases [39]. The presence of chronic pain considerably limits the autonomy of the elderly to perform daily living activities [40], which could indirectly lead to increased use of units with full care, increasing the proportion of patients with lower quality of life [41,42].

Potential limitations of our study should be addressed. For example, the cross-sectional design does not allow to characterizing a causal association, and reverse causality may play a role for some associations. Nonetheless, our findings reinforce the need for integral action in the care of elderly. Practices of health promotion are relevant to the control of chronic diseases, regardless of which one is the health problem. Increased access to PHC, offered in units with FHS, can be part of the strategy to provide assistance to people with disability and elderly above 80 years, which should an increase in the total burden of disease in the coming years.

In this study, socioeconomic status was determined by formal education and work status, but residual confounding is still possible. In fact, the socioeconomic status was positively associated with quality of life in some, but not in all countries [43]. In Canada, for example, patients have universal access to health care and socioeconomic status does not prevent to seeing a doctor. In addition, a consultation with a specialist in Canada is determined by need and not by household income, the opposite of the United States [44].

## Conclusions

The association between PHC scores and model of care reiterates the role of FHS in health care for the elderly. It also shows that the FHS contributes to higher quality of life, particularly for the mental component. These results should contribute to the management of PHC of elderly individuals.

## Competing interest

The authors declare that they have no competing interests.

## Authors' Contributions

VHSC: participated in the design of the study, performed the statistical analysis, and drafted the manuscript. SLR: participated in the study design and drafted the manuscript. FDF: participated in the study design, conceived the statistical analyses, and reviewed the manuscript. EH: conceived the study design, participated in the data analysis, and gave substantial contribution to the final version of the manuscript. SCF: conceived the study, participated in its design, performed the statistical analysis, and revised the manuscript. All authors read and approved the final manuscript.

## Pre-publication history

The pre-publication history for this paper can be accessed here:

http://www.biomedcentral.com/1471-2458/13/605/prepub
